# Antibiotic legacies shape the temperature response of soil microbial communities

**DOI:** 10.3389/fmicb.2024.1476016

**Published:** 2024-12-24

**Authors:** Carl Wepking, Jane M. Lucas, Virginia S. Boulos, Michael S. Strickland

**Affiliations:** ^1^Department of Plant and Agroecosystem Sciences, University of Wisconsin-Madison, Madison, WI, United States; ^2^Department of Biological Sciences, Virginia Tech, Blacksburg, VA, United States; ^3^Department of Soil and Water Systems, University of Idaho, Moscow, ID, United States; ^4^Cary Institute of Ecosystem Studies, Millbrook, NY, United States

**Keywords:** agroecology, carbon cycle, climate change, ecosystem function, legacy effects, livestock agriculture, microbial acclimation, microbiome

## Abstract

Soil microbial communities are vulnerable to anthropogenic disturbances such as climate change and land management decisions, thus altering microbially-mediated ecosystem functions. Increasingly, multiple stressors are considered in investigations of ecological response to disturbances. Typically, these investigations involve concurrent stressors. Less studied is how historical stressors shape the response of microbial communities to contemporary stressors. Here we investigate how historical exposure to antibiotics drives soil microbial response to subsequent temperature change. Specifically, grassland plots were treated with 32-months of manure additions from cows either administered an antibiotic or control manure from cows not treated with an antibiotic. *In-situ* antibiotic exposure initially increased soil respiration however this effect diminished over time. Following the 32-month field portion, a subsequent incubation experiment showed that historical antibiotic exposure caused an acclimation-like response to increasing temperature (i.e., lower microbial biomass at higher temperatures; lower respiration and mass-specific respiration at intermediate temperatures). This response was likely driven by a differential response in the microbial community of antibiotic exposed soils, or due to indirect interactions between manure and soil microbial communities, or a combination of these factors. Microbial communities exposed to antibiotics tended to be dominated by slower-growing, oligotrophic taxa at higher temperatures. Therefore, historical exposure to one stressor is likely to influence the microbial community to subsequent stressors. To predict the response of soils to future stress, particularly increasing soil temperatures, historical context is necessary.

## 1 Introduction

Soil ecosystems are impacted by multiple anthropogenic stressors: agricultural management and intensification (Ramirez et al., 2010; Franco et al., 2016; Vazquez et al., 2021; Wepking et al., 2022), contamination (Rillig, 2017; Wepking et al., 2017, 2019), climate (Thakur et al., 2019; Ankrom et al., 2022), and other forms of degradation (FAO, 2020). These stressors disrupt microbial communities with consequences for ecosystem functions (Strickland et al., 2009; Fierer et al., 2012; Philippot et al., 2013; Graham et al., 2016). While these stressors occur simultaneously or successively and interact over time, most soil studies examine individual factors over short timeframes (Rillig et al., 2019) limiting our understanding of how current stressors shape ecosystem responses to future disturbances. Therefore, multifactorial studies that monitor interacting stressors over time are critical (e.g., Thakur et al., 2018, 2019; Siebert et al., 2019; Lucas et al., 2021).

Temperature regulates soil activity, but anthropogenic factors push soils beyond their natural thermal range. Rising global temperatures cause variable and inconsistent alterations in soil communities and their functions (Kirschbaum, 1995, 2006; Steinweg et al., 2008; Allison et al., 2010; Conant et al., 2011; Romero-Olivares et al., 2017; Sulman et al., 2018). Warming often shifts microbial communities and metabolic pathways (Evans and Wallenstein, 2013; Luo et al., 2014; Wei et al., 2014), changes carbon turnover rates and nutrient stoichiometry in both microbial biomass and bulk soil pools (Ghee et al., 2013; Lucas et al., 2021), and impacts microbial efficiency (Schimel et al., 2007). In warmer environments, microbial C use efficiency (*CUE*) often declines (Schindlbacher et al., 2011) due to increased respiration and enzyme activity (Schindlbacher et al., 2009; Meng et al., 2020; Lucas et al., 2021). Warming-induced decreases in *CUE* may impact long-term C storage, as soil organic C pools are linked to *CUE* (Wang et al., 2021). How prior stressors affect the temperature response of soil microbial communities is not well understood.

Interacting stressors can increase the uncertainty in the response of soil processes and microbial community composition (Rillig et al., 2019), suggesting that microbial function is not driven by any one stressor. Therefore, prior environmental stressors could impact soil microbial response to future stressors. This priority effect is akin to legacy effects observed for leaf litter decomposition, where prior exposure of the soil community to a particular litter type predisposes that community to decompose a novel litter type differently (Strickland et al., 2009), or other legacy effects on soil microbial function (Osburn et al., 2022). If such priority effects exist for environmental stressors, the future response of soil microbial communities to increasing temperature could be influenced.

The introduction of antibiotics to soils via livestock waste are relatively common stressor with the potential to affect the temperature response of soil microbial communities. Antibiotic exposure can have widespread ecological consequences (Wepking et al., 2017, 2019; Grenni et al., 2018; Klein et al., 2018; McBride et al., 2020; Shawver et al., 2021). Antibiotics can shift microbial communities (Waksman, 1961; Westergaard et al., 2001; Thiele-Bruhn and Beck, 2005; Demoling et al., 2009; Reichel et al., 2013; Han et al., 2022) and their physiology (Davies et al., 2006; Wepking et al., 2017). In particular, antibiotic additions have been shown to reduce microbial biomass (Thiele-Bruhn and Beck, 2005); increase antibiotic resistance (Zhou et al., 2017; Wepking et al., 2017; Xie et al., 2018), shift microbial community composition toward more oligotrophic organisms (Markowicz et al., 2021); and increase fungal dominance (Wepking et al., 2019; Lucas et al., 2021). Additionally, antibiotic additions decrease microbial biomass C:N, and microbial *CUE* (Wepking et al., 2019, Lucas et al., 2021). This range of effects suggests that antibiotics will likely alter the response of soil microbial communities to increasing soil temperatures. Indeed, recent research indicates that antibiotics and higher temperatures interact to influence soil microbial community composition and function (Lucas et al., 2021), and also that temperature alone can increase the prevalence of antibiotic resistance (Li et al., 2022). Yet whether prior exposure to antibiotics will predispose communities to respond differently to rising temperatures is unknown.

To investigate how historical antibiotic exposure shapes soil microbial response to future warming stress, we first conducted a long-term (32-month), common-garden, field experiment. Field plots received monthly manure additions from cattle either administered or not administered one of two types of antibiotics (cephapirin and pirlimycin) as described in Wepking et al. (2019). We predicted that initially soils amended with manure from antibiotic-treated cattle would respire C at an elevated rate compared to manure alone with the largest differences seen when soil temperatures were at their greatest, but this response would attenuate with time due to antibiotic induced shifts in microbial community composition and function. At the conclusion of the field experiment, soil samples were collected and incubated across a temperature range (15, 20, 25 and 30°C) to determine how prior antibiotic exposure influences soil’s response to warming. Because cephapirin and pirlimycin both have the potential to reduce microbial biomass, growth efficiency, and lead to shifts toward a more oligotrophic community, we expected that both could induce an acclimatory-like response to temperature when compared to the control. Alternatively, prior exposure to antibiotics may cause mass mortality of microbiota leading to reduced substrate availability and shifts in C cycling pathways over time. If prior antibiotic exposure elicits temperature acclimation or shifts in substrate availability, then this could influence predictions related to soil warming across agricultural landscapes and highlights the potential that legacy effects associated with environmental stressors may influence the response of soils to future unrelated stressors.

## 2 Methods and materials

### 2.1 Field experiment

This experiment consisted of a three-year field-based common garden experiment, followed by a laboratory-based incubation experiment (see Supplementary Table S1 for experimental replication information). This field experiment continued the manure addition protocol from Wepking et al. (2019). Briefly, three cattle manure treatments – from cattle administered one of two types of antibiotics cephapirin benzathine (hereafter *Ceph*; ToMORROW®; Boehringer Ingelheim Vetmedica, Inc., Duluth, GA, USA; intramammary dry cow therapy; single 300-mg dose into each quarter), or pirlimycin hydrochloride (hereafter *Pir*; Pirsue®; Zoetis, Parsippany, NJ, USA; intramammary dose standard for clinical mastitis; two 50-mg doses, 24-h apart), or cattle not administered antibiotics (i.e., control manure; hereafter *Con*) – were applied to field plots. Both antibiotics were selected as they are commonly used for prophylactic treatment of mastitis in dairy cows. Manure was applied monthly over the course of the 32-months (total 21,000-g m^−2^; 648-g m^−2^ month^−1^ of wet-weight manure, corresponding with a typical stocking density). For further information, including information on manure characteristics, and antibiotic residual detected within manure, please see Wepking et al. (2019).

Soil respiration was measured monthly over the course of the 943-d field experiment using a LI-8100A infrared gas analyzer (IRGA) and 8100–103 20-cm Survey Chamber (LI-COR Biosciences, Lincoln, Nebraska, USA). Environmental data (soil temperature, air temperature, precipitation) was collected from the Kentland Farm Weather Station. In order to examine the interactive effects of temperature and antibiotics, Q_10_ (Kirschbaum, 1995) – a measurement of expected change for a given parameter with a 10°C change in temperature – was determined for each experimental plot using soil respiration and corresponding temperature measurements. The dependence of soil respiration on temperature was described by the equation:

$Y_{T}=Be^{kT}$

where *Y_T_* is soil respiration (μmol m^2^ s^−1^), *T* is temperature (°C), *B* is the exponential fit parameter for the intercept, and *k* is the exponential fit parameter for the slope. For each experimental plot we then calculated Q_10_ using the following equation:

$Q_{10}=e^{10k}$

The total respiration from the field experiment was calculated via integration.

### 2.2 Incubation experiment

Soil was collected from each plot within the randomized block, common garden experiment described above (three treatments, six blocks). While this experiment is described in greater detail in Wepking et al. (2019), briefly, soil was collected in 0.05 m^2^ subplots to a depth of 10-cm. Each monolith was broken down into component parts – soil, roots, and aboveground plant material. The most recent manure application was one month prior to the soil sampling, therefore little manure remained visible within the plots. Following sieving (4.75-mm) and homogenization, eight grams (dry weight equivalent) of soil was placed in 50 mL centrifuge tubes, modified to allow for gas sampling. As these source soils came from a randomized block experiment (three treatments, *n* = 6), this block format was maintained in this study. A technical replicate was also added for each iteration of block and treatment. Soils were maintained at 65% water holding capacity.

### 2.3 Carbon mineralization

Soils were incubated for 60-d at four temperatures (15°C, 20°C, 25°C, and 30°C) following a 24 h pre-incubation at these same temperatures. During this time carbon mineralization (hereon, *Cmin*) was determined bi-weekly for the first two weeks, to better capture the initial peak of carbon respiration, and then weekly for the rest of the experiment. Respiration was determined with an infrared gas analyzer (IRGA; Model LI-7000, LI-COR Biosciences, Lincoln, NE, USA) using a static incubation technique (Fierer et al., 2003). Total respiration was calculated via integration.

### 2.4 Active microbial biomass

Active microbial biomass (substrate-induced respiration; *SIR*; West and Sparling, 1986, Fierer et al., 2003) was determined on pre-and post-incubation soils to detect changes in microbial biomass over the course of the incubation (*∆SIR*). SIR was determined following Wepking et al. (2017); see Supplementary methods for more information. Prior to incubation, additional assays of soil function and edaphic properties were conducted (i.e., pH, *SIR*, standard *Cmin* at 20°C; Supplementary Table S2).

### 2.5 Mass specific respiration and microbial community analyses

Antibiotic exposure (Wepking et al., 2017) and increased temperature (Frey et al., 2013) alter microbial physiology, with reduced microbial efficiency being observed in response to both disturbances. Therefore, to better understand microbial efficiency, mass-specific respiration (*MSR*) was calculated and analyzed (Wepking et al., 2017). This consisted of the total C mineralized as a proportion of the total microbial biomass as determined by SIR. The microbial biomass (*SIR*) used for this analysis was an average of starting and ending microbial biomass.

Following the 60-d incubation, 0.5-g samples of soil were frozen at −80°C to determine the microbial community response. We amplified ribosomal marker genes using 2 step PCR in accordance with the Earth Microbiome Project protocol for *16S* and ITS sequencing (The Earth Microbiome Project Consortium et al., 2017). See Supplementary methods for additional details.

### 2.6 Statistics

Data analysis was performed with the R statistical platform (R Core Team, 2017). Linear mixed models (LMM) — ‘lme4’ package (Bates et al., 2015) – were used to create linear models which were analyzed using analysis of variance (ANOVA). Model reduction analysis was used to examine the influence of temperature and treatments. Model selection was determined by lowest Akaike information criterion (AIC) score (Akaike, 1974). For the field experiment, treatment was a fixed effect and block was a random effect. For the incubation experiment, treatment and temperature were interactive fixed effects and block was a random effect. Variance was tested with a Wilk–Shapiro test. Where assumptions were not met data were log or square-root transformed.

Microbial community structure was compared with Primer (Ver. 7.0.13) and R (package *vegan;* Oksanen et al., 2020). Microbial community data was square-root transformed before calculating Bray–Curtis dissimilarity. Community distance matrices were used to generate ordinations for bacteria and fungi. To compare community composition across treatment and temperature as well as interactive effects PERMANOVA was used (Anderson, 2001; Anderson et al., 2008), and beta diversity was assessed across our samples using PERMDISP tests (Anderson, 2006; Anderson et al., 2006). Linear mixed effects models were used to determine how the most abundant bacterial and fungal orders differed across each temperature. Because of the significant interaction between treatment and temperature in our PERMANOVA analysis, we subsetted our data and examined the treatment effect at each incubation temperature.

## 3 Results

### 3.1 Field-measured microbial respiration

**Figure 1:**
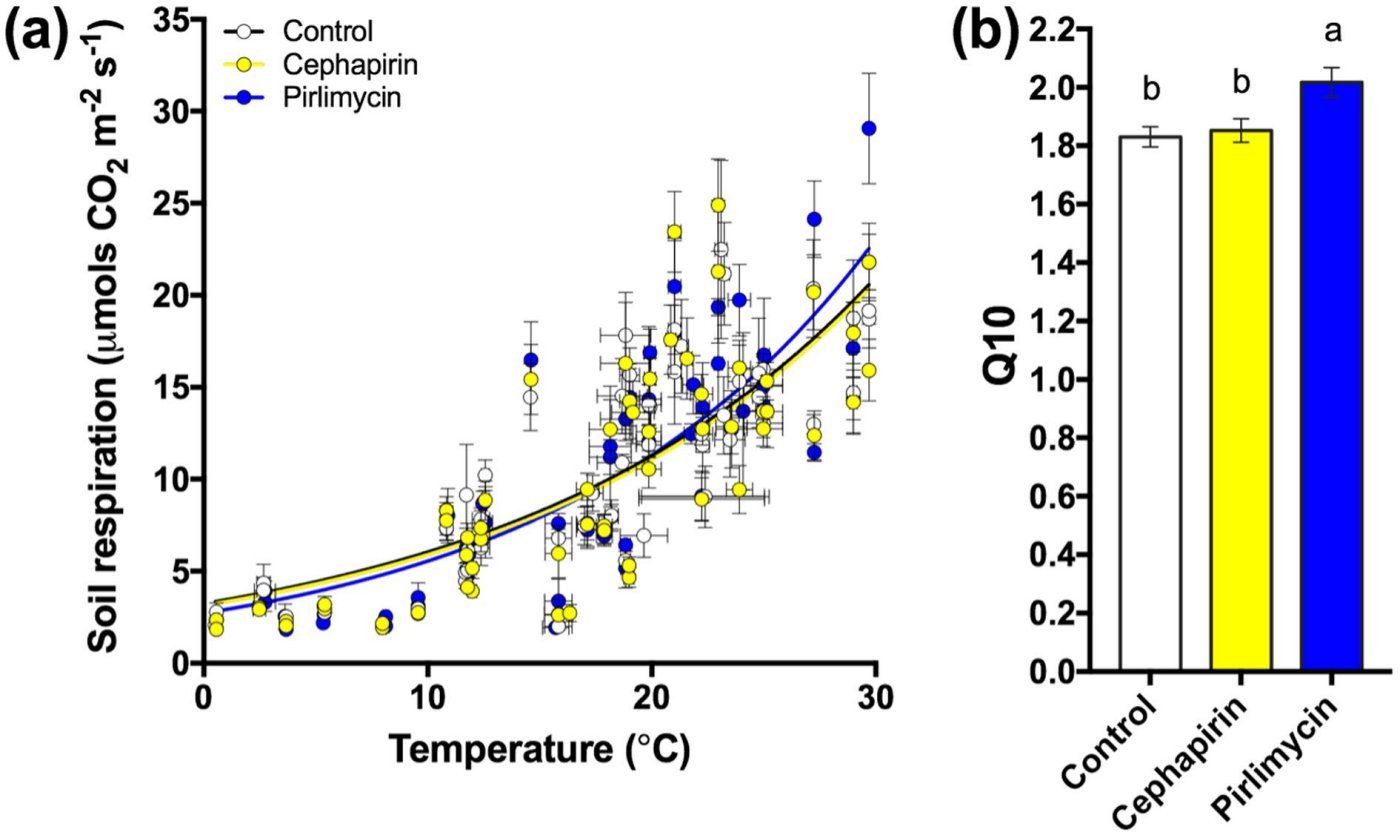
Summary of the response of the treatments to changing temperature during 32-months of manure additions. **(A)** Monthly measurements of soil respiration across 32-months and their relationship to air temperature. **(B)** Treatments vary by Q_10_ quotient with the *Pir* treatment respiring more carbon at higher temperatures than the other two treatments.

**Figure 2:**
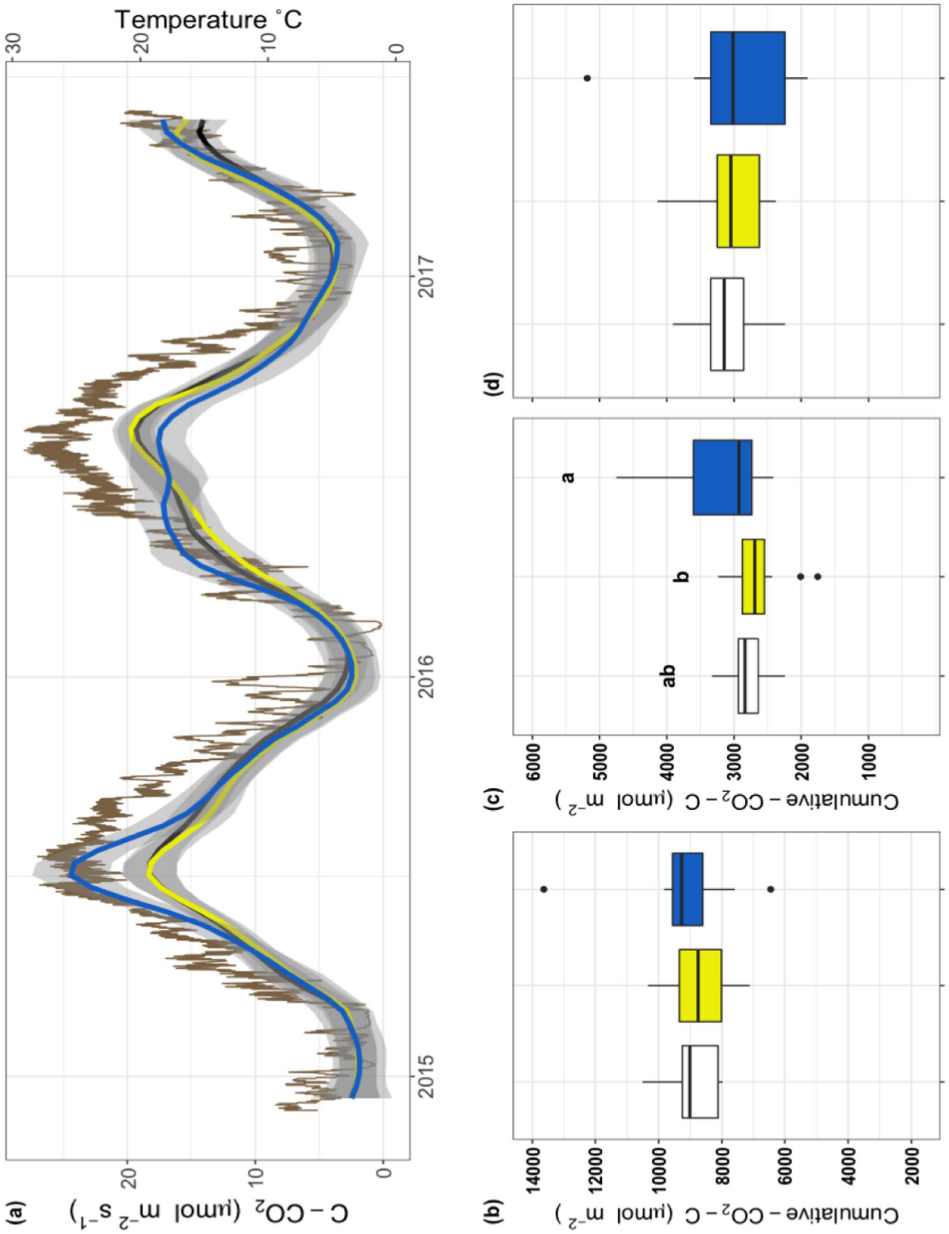
Field respiration and soil temperature over the course of the three-year experiment. **(A)** Field respiration from December 2015 through May 2017. Respiration of *Con* (black), *Ceph* (yellow), and *Pir* (blue) are shown as smoothed lines with gray shaded errors; the scale for this data is on the left y-axis. Standard error was calculated using *t*-based confidence intervals. The brown line depicts soil temperature over this sampling period; the scale for this data is on the right y-axis. **(B–D)** Cumulative respiration for the three treatments, *Con* (white), *Ceph* (yellow), and *Pir* (blue), as determined by calculating the integral of field respiration during 3 periods of time; **(B)** over the course of the entire 32-month experiment; **(C)** during the 2015-growing season; and **(D)** during the 2016-growing season.

To establish whether there were antibiotic effects on soil microbial respiration we compared the relationship between soil temperature and respiration during the 32-month field-portion of the experiment (Figure 1A). Additionally, to explore interactive effects of temperature with antibiotic exposure, we calculated the coefficient Q_10_ and subsequently analyzed it across the entire field experiment (Figure 1B); a significant treatment effect was observed with the *Pir* treatment having a higher Q_10_ than the control or the *Ceph* treatment (*F_2,10_* = 4.56, *p* < 0.05). Subsequently, we considered the field-measured respiration as a timeseries (Figure 2A) as well as a cumulative total (Figure 2B), the latter not indicating a significant treatment effect (*F_2,28_* = 0.47, *p* = 0.63). However, when examining the data on a finer scale, treatment differences during the warmer months of the year are apparent (Figure 2A).

To better understand the warm season patterns, each growing season was analyzed individually. To account for inter-year variability in growing season, temperature was used as a proxy. Data were partitioned into peak growing periods during 2015 and 2016 by isolating respiration measurements taken when soil temperature exceeded 12°C. This cutoff was based on previous studies of grass showing a divergence in growth rate when the temperatures of soils were between 10°C and 15°C (Delucia et al., 1992). Cumulative respiration in the growing season of 2015 showed a significant treatment effect (Figure 2C; *F_2,29_* = 4.05, *p* < 0.05), while no significant treatment effect was observed during the 2016 growing season (Figure 2D; *F_2,33_* = 0.24, *p* = 0.79). Within the 2015 growing season (Figure 2C), the treatment effect was driven by the Pir treatment which was greater than *Con* (*p* = 0.051) and significantly greater than *Ceph* (*p* < 0.05).

### 3.2 Incubation experiment

#### 3.2.1 Active microbial biomass

**Figure 3:**
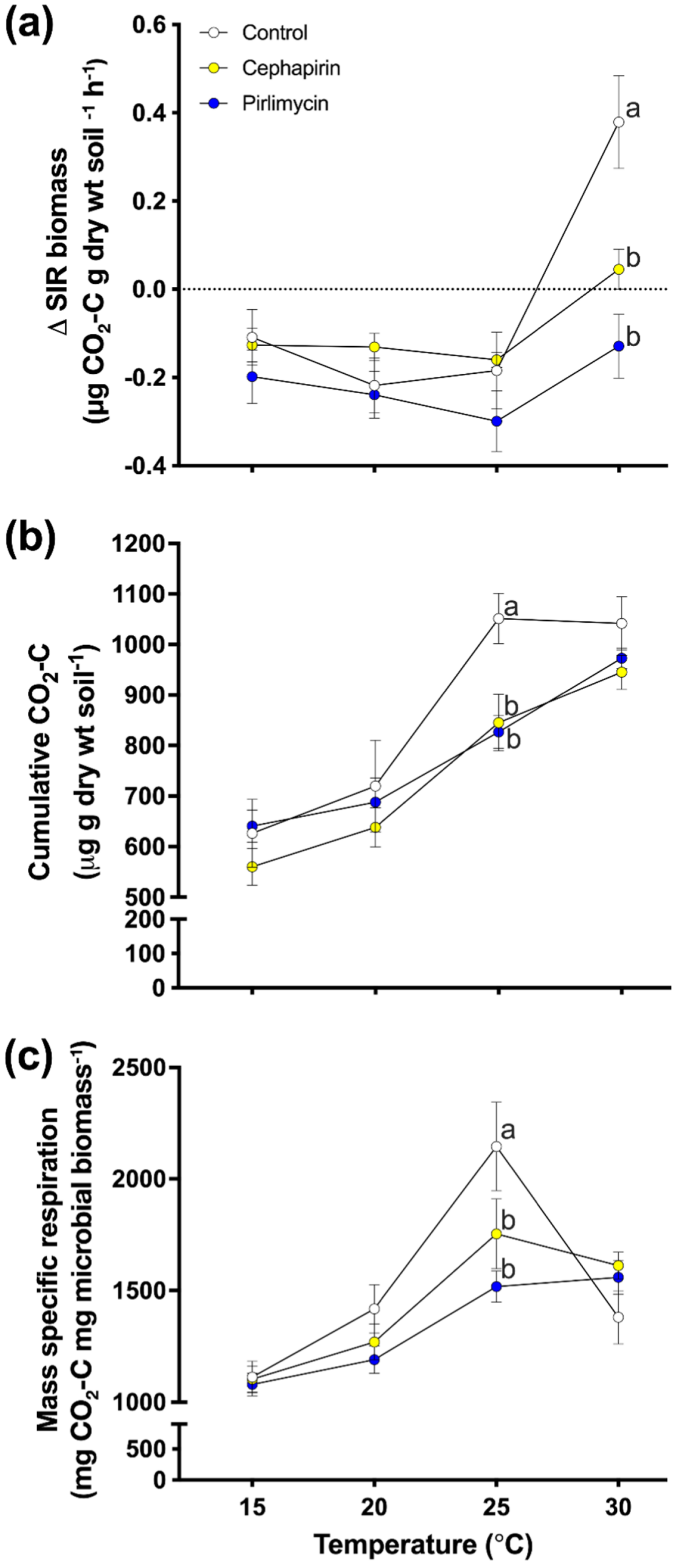
Response of treatments to a 60-d incubation at a range of temperatures (15°C, 20°C, 25°C, 30°C). **(A)** Change in active microbial biomass (*∆SIR*; μg CO_2_-C g dry wt soil ^−1^ h^−1^) from the beginning to the end of the incubation. Within temperatures, there was a significant difference between treatments at the 30°C temperature (*Con* was significantly greater than *Ceph* and *Pir* antibiotic treatments). **(B)** Cumulative amount of microbially respired carbon (*Cmin*) across the three treatments and four temperatures. For cumulative respired carbon, the 25°C treatment was the only temperature to show significant treatment effects (*F_2,28_* = 9.10, *p* < 0.001; Con significantly greater than *Ceph* and *Pir* antibiotic treatments). **(C)** The amount of respired carbon as a proportion of microbial biomass for the incubation experiment. For mass-specific respiration **(C)**; mg CO_2_-C mg microbial biomass^−1^, the 25°C treatment was the only temperature to show a significant treatment effect (*F_2,28_* = 9.10, *p* < 0.001; Con significantly greater than *Ceph* and *Pir* antibiotic treatments).

The final active microbial biomass (i.e., post-incubation) and the change in active microbial biomass (*∆SIR*; Supplementary Table S3) showed similar dynamics, therefore we have focused on the *∆SIR*. There were significant main effects of treatment (Figure 3A; *F_2,127_* = 14.35, *p* < 0.001), temperature, (Figure 3A; *F_3,127_* = 25.82, *p* < 0.001), and a treatment × temperature interaction (Figure 3A; *F_6,127_* = 5.87, *p* < 0.001). The temperature effect was largely driven by an increase in *SIR* in the 30°C incubation; all other temperatures showed a decrease in *SIR*. Within temperatures, the control treatment had a higher *∆SIR* than the two antibiotic treatments in the 30°C incubation.

#### 3.2.2 Carbon mineralization

Analysis of *Cmin* throughout the entire incubation (Supplementary Table S4) revealed significant treatment (Figure 3B; *F_2,127_* = 6.66, *p* < 0.01) and temperature effects (Figure 3B; *F_3,127_* = 48.44, *p* < 0.001) but no treatment × temperature interaction (Figure 3B; *F_6,127_* = 1.38, *p* = 0.23). Overall, the treatment effect is attributed to the antibiotic treatments mineralizing less C than the control across temperatures. The temperature effect was largely as predicted, with each 5°C increase creating a significant increase in *Cmin* (apart from 15°C and 20°C which was only a marginally significant difference; *p* = 0.059). When examined within incubation temperatures, no significant treatment effect is noted at 15°C (Figure 3B; *F_2,28_* = 1.09, *p* = 0.35), 20°C (Figure 3B; *F_2,28_* = 0.52, *p* = 0.60), or 30°C (Figure 3B; *F_2,28_* = 2.0, *p* = 0.16). However, a significant treatment effect is observed in the 25°C incubation (Figure 3B; *F_2,28_* = 9.10, *p* < 0.001). This was primarily driven by reduced *Cmin* in the *Ceph* (*p* < 0.01) and *Pir* (*p* < 0.001) treatments compared to the control.

#### 3.2.3 Mass specific respiration

Analysis of *MSR* (Supplementary Table S5) from the incubation experiment showed a marginally significant treatment effect (Figure 3C; *F_2,127_* = 2.62, *p* = 0.08), and a significant temperature (Figure 3C; *F_3,127_* = 44.02, *p* < 0.001) and treatment × temperature interaction (Figure 3C; *F_6,127_* = 3.87, *p* < 0.005). *MSR* was greatest in the 25°C incubation compared to all other incubations (all: *p* < 0.005). The 30°C incubation showed the second highest *MSR* and was significantly greater than both the 15°C (*p* < 0.001) and 20°C (*p* < 0.001) incubations. Finally, *MSR* of the 20°C incubation was significantly greater than that of the 15°C incubation (*p* < 0.005). The marginal treatment, and significant treatment × temperature interaction effect was largely driven by the dynamics within the 25°C incubation. Here, *Con* showed a significantly greater *MSR* than both *Ceph* (*p* < 0.005) and *Pir* (*p* < 0.001). There were no other significant treatment effects within the other incubation temperatures.

#### 3.2.4 Microbial community composition

Shannon diversity (H) – a measurement of richness and evenness – was influenced by both treatments and temperature for the fungal communities, while the bacterial communities were unaffected by either. Fungal Shannon diversity increased in both antibiotic treatments compared to controls (dAIC = −34.12, χ^2^_3_ = 40.12, *p* < 0.001, Supplementary Figure S1). However, as temperatures increased, fungal diversity decreased (dAIC = −14.89, χ^2^_3_ = 18.89, *p* < 0.001). Specifically, 15°C and 20°C environments had high fungal diversity, with 25°C having lower and 30°C having the lowest (Supplementary Figure S1). There was no interactive effect of temperature and treatment on fungal (dAIC = 6.53, χ^2^_6_ = 5.47, *p* = 0.49) or bacterial Shannon diversity (dAIC = 2.91, χ^2^_6_ = 9.09, *p* = 0.17).

**Figure 4:**
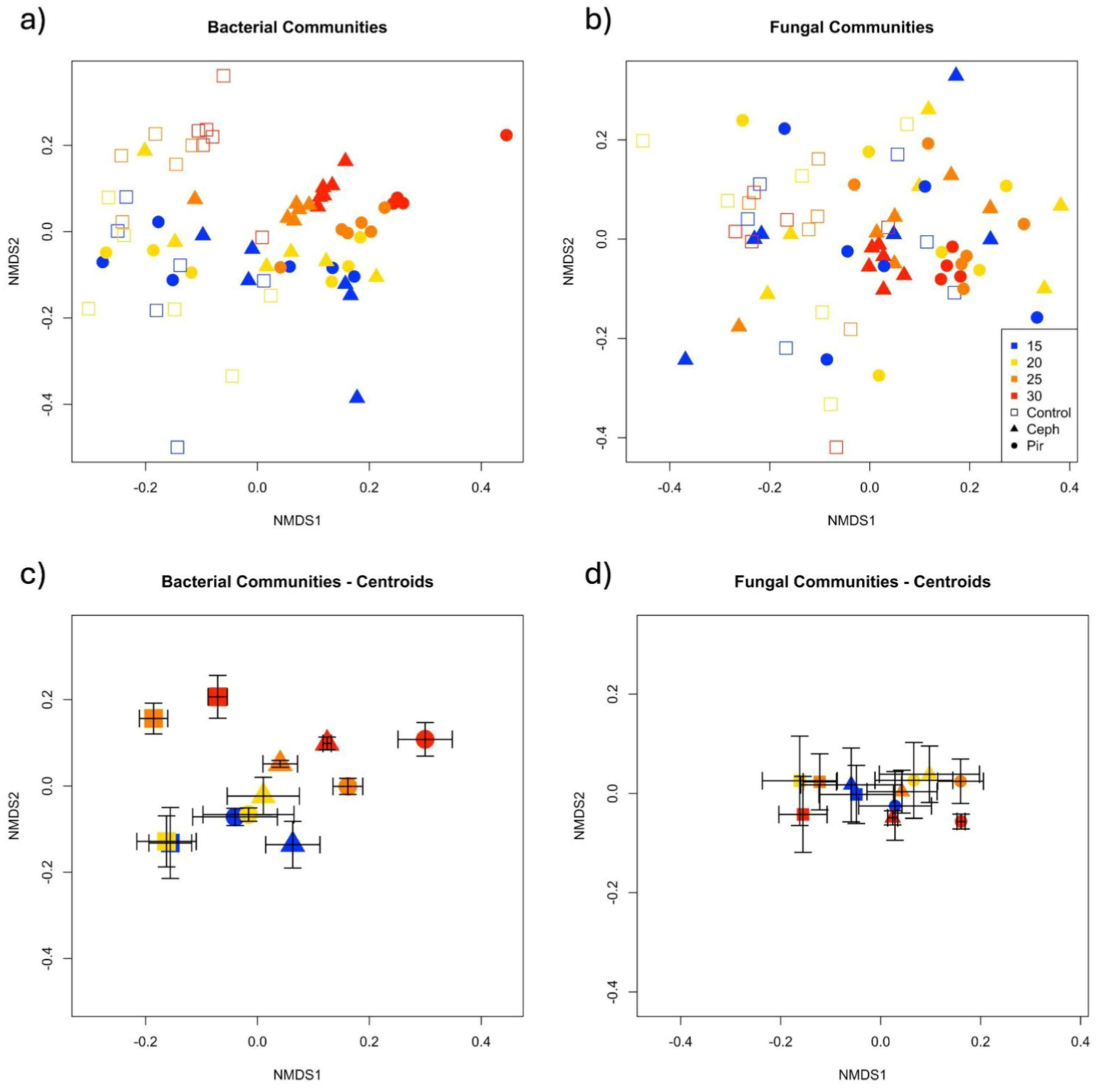
Non-metric dimensional scaling representation of **(A,C)** bacterial and **(B,D)** fungal communities associated with temperature and antibiotic treatments. Distances are based on dissimilarity matrices of sequence-based Bray-Curtis distances. Panels **(C,D)** represent the centroid for each antibiotic and temperature treatment, and error bars are mean standard error.

The composition of microbial communities responded to both temperature and antibiotic treatments during the 60-day incubation (Figure 4; Supplementary Figure S2; Supplementary Tables S4, S5). Both fungal and bacterial communities were influenced by antibiotic treatments (PERMANOVA Fungi: Figure 4, *Pseudo-F_2,53_* = 4.93, *p* < 0.001; Bacteria: Figure 4A, *Pseudo-F_2,51_* = 4.69, *p* < 0.001), temperature incubations (PERMANOVA Fungi: *Pseudo-F_3,53_* = 1.94, *p* < 0.001; Bacteria: *Pseudo-F_3,51_* = 3.21, *p* < 0.001), and their interaction (PERMANOVA Fungi: *Pseudo-F_6,53_* = 1.3, *p* < 0.001; Bacteria: *Pseudo-F_6,51_* = 1.52, *p* < 0.001). Temperature effects on bacterial and fungal composition were partially driven by a decrease in beta diversity at higher temperatures (PERMDISP Fungi: Pseudo-F3,66 = 13.04, *p* < 0.001; Bacteria: Pseudo-F3,64 = 3.64, *p* = 0.049).

Microbial communities were analyzed within each temperature separately to examine temperature × treatment interaction; treatment effects were only apparent in soils maintained at 25°C and 30°C (Table 1). Specifically, at both 25°C and 30°C, *Pir* and *Ceph* differed from each other and from controls. To further examine this trend we looked at the relative abundance of the top 10 most abundant bacterial orders across treatments (Supplementary Table S4; Supplementary Figure S2). We found that community differences at 25 and 30°C were driven by increases in Acidobacteriales (phylum Acidobacteriota), Pedoshaerales (phylum Verrucomicrobia), and Chthoniobacterales (phylum Verrucomicrobia) in antibiotic treatments (Supplementary Table S4; Supplementary Figure S2). Additionally, Micropepsales (phyla Proteobacteria) increased in relative abundance at 15, 25 and 30°C in antibiotic plots compared to controls. Myxococcales (phylum Myxococcota) decreased in relative abundance in *Pir* plots compared to *Ceph* at 25°C, and Betaproteobacteria (phylum Proteobacteria) decreased in antibiotic plots at 30°C (Supplementary Table S4).

**Table 1:** Results of the PERMANOVA analysis of microbial communities.

Bacterial community	Fixed effect	Subsetted-model	*Pseudo-F*	d.f.	*p*-value
Full model	Treatment × Temperature		1.52	6,51	**0.0001**
	Treatment		4.69	2,51	**0.0001**
	Temperature		3.21	3,51	**0.0001**
Temperature subsets	15	Treatment	1.07	2,10	0.36
	20	Treatment	1.11	2,10	0.3
	25	Treatment	3.73	2,9	**0.0003**
	30	Treatment	4.38	2,8	**0.0004**
Fungal community					
Full model	Treatment × Temperature		1.3	6,53	**0.0009**
	Treatment		4.93	2,53	**0.0001**
	Temperature		1.94	3,53	**0.0001**
Temperature subsets	15	Treatment	1.01	2,10	0.5
	20	Treatment	1.1	2,10	0.32
	25	Treatment	3.46	2,10	**0.0002**
	30	Treatment	5.1	2,8	**0.0002**

Initial full models were run to examine the influence of fixed effects and their reactions. Because of a significant interaction between treatment and temperature, we subsetted our data and examined the treatment effect at each incubation temperature. The results of these models are provided below the full model section. Bold values indicate statistical significance.

Fungal communities also differed across treatments at high temperatures (25 and 30°C, Supplementary Table S5). As seen in previous work, fungal communities became more homogenous at high temperatures (Lucas et al., 2021), and shifted in composition. The most prominent difference between antibiotic treatments and controls was the high relative abundance of Microascales in Controls at 25 and 30°C (Supplementary Table S5). Ceph and Pir soils also had increased relative abundance of Mortierellales at 25 and 30°C. In Pirlimycin soils, the relative abundance of Archeaorhizomycetales and GS11 increased and Sordariales decreased at 25 and 30°C, and Olipidales increased at 25°C only (Supplementary Table S5).

## 4 Discussion

Antibiotic exposure caused soil respiration to respond differently to seasonal temperature shifts during the first year of the field experiment. That is, the *Pir* treatment exhibited a greater increase in respiration at higher temperatures, whereas the *Ceph* treatment tended to elicit less of an increase in respiration. However, after prolonged exposure to antibiotics, these effects diminished suggesting an acclimatory-like response to temperature variation. To examine whether this legacy of antibiotic exposure affects the temperature response of the soil microbial communities, we incubated soils from our field experiment across a range of temperatures. We found that antibiotic legacies (versus the control) led to a divergence in the response of microbial communities at warmer temperatures (25 and 30°C). Soils previously exposed to antibiotics exhibited a decrease in SIR and CMIN at warmer temperatures, suggesting that response to a current stressor (here, warmer temperatures), may be shaped by legacy stressors (here, antibiotic exposure).

During the first year of the field experiment, the *Pir* treatment was associated with a greater temperature response for soil respiration when compared to the control or *Ceph* treatments. It is perhaps unsurprising that the *Ceph* treatment does not exhibit a similar respiratory response because the two antibiotics vary in their modes of action and therefore differentially effect microbial community structure and physiology (Lobritz et al., 2015; Wepking et al., 2017, 2019). Antibiotic mode of action also has practical impacts related to their potential ecological effects, e.g., bacteriostatic antibiotics, like pirlimycin, inhibit bacterial growth (Lobritz et al., 2015). Therefore, we predicted that as temperature increased, respiration in *Ceph* treatments would likely be lower due to cell death. In contrast, bacteriostatic antibiotics such as pirlimycin should increase respiration due to decreased microbial efficiency (Lobritz et al., 2015, Wepking et al., 2017, 2019). Additionally, previous research at this site found both a shift toward greater fungal-dominance, as well as, compositional differences in both bacterial and fungal communities across the entire experimental period (Shawver et al., 2021) when comparing the antibiotic treatments to the control. Such shifts in microbial community structure – whether attributed to a direct effect of the antibiotic, or an indirect effect of the manure microbiome on the soil microbiome, or a combination of the two – could cause varied respiratory responses to temperature (e.g., Pietikäinen et al., 2005).

Interestingly, differences in the temperature response of the antibiotic treatments attenuated over time, similar to previous soil warming experiments (Bradford, 2013), where microbial communities down-regulate activity under sustained high temperatures. However, in this instance the acclimation-like response was not driven by exposure to higher or lower temperatures but was in fact due to historical antibiotic exposure. We suggest that microbial communities exposed to antibiotics had to initially up-regulate metabolically expensive defensive strategies (e.g., antibiotic resistance), leading to elevated CO_2_ (Liu et al., 2009). However, in subsequent years, the community composition and functional capacity to endure antibiotic exposure, allowed microbial communities to maintain respiration rates similar to controls, i.e., acclimate. Additionally, this acclimation-like response was likely not driven by changes in soil C availability or microbial biomass as can occur in soil warming experiments (Conant et al., 2011; Bradford, 2013). We did not observe differences in bioavailable-C or microbial biomass at the end of the field experiment (Supplementary Table S2). When exposing our field soils to glucose for pre-incubation SIR measurement, the response was similar across treatments, suggesting a lack of substrate limitation. Instead, differences in microbial community composition and/or physiology are likely accounted for by this acclimatory-like temperature response.

Legacy antibiotic exposure shaped the temperature response of these soils. Specifically, microbial biomass, soil respiration, and mass-specific respiration all exhibited reduced response to increasing temperatures for the antibiotic-exposed soils compared to the control. Additionally, temperature driven shifts in the microbial community were contingent on antibiotic exposure. There are a number of potential explanations for the observed temperature dependent effects, including a shift in thermal optima (Bradford, 2013), or decreased microbial efficiency due to microbial stress (Schindlbacher et al., 2011). It is also possible that substrate availability became limiting in control soils at 30°C. In control environments, C mineralization rates stabilized between 25 and 30°C, and microbial activity was stimulated by the addition of glucose from SIR assays at 30°C, possibly indicating substrate limitation. However, substrate limitation was not likely a factor in antibiotic laden environments as C mineralization rates increased with increasing temperature, and microbial activity was low even when glucose was added. Combined, the varied microbial dynamics between antibiotic-exposed soils and control soils emphasizes how historical stress can lead to divergent future trajectories.

At 30°C active microbial biomass associated with the control increased significantly more than either antibiotic treatment (both: *p* > 0.005), aligning with expectations for recently warmed soils. In contrast, the *Ceph* treatment was found to have a marginally greater increase in microbial biomass than *Pir* (*p* = 0.08) providing evidence that these soils – despite all being exposed to additional resources in the form of manure – do not function similarly with increasing temperature even after 32-months of manure additions. Instead, the antibiotic treatments exhibited reduced microbial growth (or activity) similar to thermally acclimated microbial communities (Bradford et al., 2008).

Divergence in both cumulative respiration and *MSR* at a moderate-high temperature and convergence at both low and high temperatures appears to emulate the Type III response of microbial acclimation described by Bradford et al. (2008) and Bradford (2013), driven by a shift in thermal optima of the antibiotic-exposed microbial communities. This response is likely attributable to either microbial turnover or acclimation. The 25°C incubation, where we saw a divergence in cumulative respiration and *MSR*, was also the warmest temperature *not* to show an increase in microbial biomass over the course of the incubation. Instead, it shows the greatest respiration per unit biomass – this is driven by the control, as the two antibiotic treatments are not significantly different from the 30°C incubation. It is possible that a shift in bacterial-to fungal dominance could drive this concomitant shift in thermal optima, given that fungi are inhibited with increasing temperature relative to bacteria (Pietikäinen et al., 2005). Such a shift in fungal dominance was observed in the field (Wepking et al., 2019). Subtle changes in microbial and fungal community composition could also play a role.

In fact, for soil microbial communities we observed a difference in temperature response based on antibiotic legacy. Specifically, at lower temperatures microbial communities were compositionally more similar but at higher temperatures (i.e., 25 and 30°C) microbial communities were compositionally more dissimilar among treatments and exhibited lower within treatment beta diversity. Previous studies have shown that exposure to antibiotic-laden manure can drive changes in microbial community composition (Wepking et al., 2019; Shawver et al., 2021). Surprisingly, in this study we found that past exposure to antibiotics shapes the compositional response of the soil microbial community to increasing temperature. In fact, it is this compositional response that may drive the acclimatory-like response to temperature. Specifically, we observed that oligotrophic microbial taxa (i.e., taxa in the phylum Acidobacteria and Verrucomicrobia) and many fungal saprobes (i.e.*, Fusarium, Humicola*) increased in relative abundance to a greater extent at higher temperatures for the antibiotic-exposed soils. This shift toward oligotrophic bacterial dominance – with their lower growth rates and higher growth yield efficiency (Fierer et al., 2007) – likely accounts for the varied temperature response. This shift toward a more oligotrophic community was not ultimately a product of exposure to extended high temperatures or low substrate availability, instead it was a product of historical antibiotic exposure.

Exposure to antibiotics can shape the thermal response of soil microbial communities. This acclimatory-like response to increasing temperatures suggests that the temperature response of soils exposed to antibiotics may be less significant than un-exposed soils. Further research is needed to better understand the entirety of antibiotic effects on soil communities and function. For instance, whether soils from a range of environments respond in a similar manner (Wepking et al., 2017); if the observed initial decrease in whole ecosystem *CUE* caused by antibiotic exposure offsets any potential benefit derived from a change in temperature response (Wepking et al., 2019); and whether continued, simultaneous exposure to antibiotics and higher temperatures leads to greater losses of soil C (Lucas et al., 2021). Additionally, this research introduces the underexplored concept of historical legacies in soils and emphasizes the potential for past stress to impact the response of soil communities to future stressors. To date, few studies have examined how multiple stressors can simultaneously influence soil community structure and function (Rillig et al., 2019), fewer if any have considered legacy effects. To fully understand the response of soils to future conditions we must consider both their present state, and the historical legacies that shaped these soil communities.

## Data Availability

The datasets presented in this study can be found in online repositories. The names of the repository/repositories and accession number(s) can be found at: https://datadryad.org/stash, doi: 10.5061/dryad.dncjsxm7t.
