# Supplementary material for: Antibiotic legacies shape the temperature response of soil microbial communities
Source: Front Microbiol. 2024 Dec 24;15:1476016. doi: 10.3389/fmicb.2024.1476016 (PMC11703895; doi:10.3389/fmicb.2024.1476016)
Supplement: Supplementary file 1 [file Data_Sheet_1.docx]

Journal Name: Frontiers in Microbiology

Manuscript Type: Research Article

Manuscript Title: Antibiotic legacies shape the temperature response of soil microbial communities

Running Title: Antibiotics & microbial temperature response

Authors: Carl Wepking^a,b^, Jane M. Lucas^c,d^, Virginia S. Boulos^b^, Michael S. Strickland^c^

Affiliations: ^a^Department of Plant and Agroecosystem Sciences, University of Wisconsin-Madison, Madison, WI, 53706 *(CW present address)*

^b^Department of Biological Sciences, Virginia Tech, Blacksburg, VA 24061

^c^Department of Soil and Water Systems, University of Idaho, Moscow, ID 83844

^d^Cary Institute of Ecosystem Studies, Millbrook, NY 12545 *(JML present address)*

Supplementary Materials:

Supplementary Methods:

Determination of Active Microbial Biomass:

The process for active microbial biomass via SIR was as follows: 4-g of dry weight equivalent soil was incubated for 24-h at 20˚C; an autolyzed yeast substrate (792-mg of yeast in dissolved 8-ml of DI water) was added to each sample (this quantity of yeast substrate exceeds microbial demand so that differences in amount respired are due entirely to the amount of active microbial biomass); samples were then homogenized with a vortexer and shaken for 1-h; samples were capped and the headspace flushed with CO_2_-free air and then returned to the 20˚C incubator for 5-h; finally, the microbially respired CO_2_ that has accumulated in the headspace of each sample was determined using a gas syringe and a bench-top infrared gas analyzer (IRGA, LI-7000 CO_2_ H_2_O Analyzer, Li-Cor, Lincoln, NE).

Microbial Community Analyses:

At the end of the 60-d incubation period, 0.5-g samples of soil were frozen at -80˚C to determine the microbial community response to antibiotic additions and temperature. We amplified ribosomal marker genes using 2 step PCR in accordance with the Earth Microbiome Project protocol for 16S and ITS sequencing (The Earth Microbiome Project Consortium et al., 2017). We used the ITS1F/ITS2 and the 515f/806r primer pairs for fungi and bacteria, respectively. After the first round of PCR, sequences were cleaned using ExoSAP-ITTM PCR cleanup reagent (Affymetrix Inc., Santa Clara, CA, USA), according to the manufacturer’s protocol. During the second round of PCR, we attached unique barcoded primers to each sample. After the second round of PCR, samples were cleaned and normalized using SequelPrepTM 96-well plates (Invitrogen, Carlsbad, CA, USA). We pooled equimolar DNA and sequenced these amplicon pools on an Illumina MiSeq instrument using 2 × 300 bp sequencing kits at the IBEST sequencing facility at the University of Idaho. We used controls throughout the laboratory process to ensure there were no contaminants. Raw sequence data are available at FigShare (doi: 10.6084/m9.figshare.16722958).

Raw sequences were first demultiplexed by the IBEST genomic resource core using the program dbcAmplicons (Uribe-Convers et al., 2016). This process also removed barcodes and primers from sequences. We then processed paired sequences using the DADA2 pipeline (Callahan et al., 2016), which is designed to resolve exact biological sequences from Illumina sequence data and does not involve sequence clustering (Leff et al., 2018). Paired sequences were trimmed to uniform lengths, dereplicated, and the unique sequence pairs were denoised using the ‘dada’ function, accounting for errors through the model generated with the ‘learnErrors’ command. We merged these paired-end sequences and removed chimeras. We assigned taxonomy using the Silva database (ver. 132, Quast et al., 2013) and the UNITE dynamic general release (ver 01.12.2017; Abarenkov et al., 2010) databases for bacteria and fungi, respectively. We filtered out sequences found in negative controls, as well as sequences coding for mitochondria and chloroplast using the mctoolsr package (Leff, 2016) using R software (R Core Team, 2017). To account for differences in sequencing depths, we rarefied samples to 3010 and 1574 sequences per sample for fungi and bacteria, respectively. This required us to drop one bacterial sample from our 15˚C control plot due to poor sequence quality.

Supplementary Table 1:

| Scale of inference | Scale at which the factor of interest is applied | Number of replicates at the appropriate scale |
| --- | --- | --- |
| Block (Field) | Plot (Field) | 6 |
| Field plot soils nested in blocks | Microcosms at four temperatures | 3 plots per block (6 blocks); 2 analytical replicates per microcosm |

Table S1: Replicability statement.

Supplementary Table 2:

(Table S2):

|  | pH | *Cmin* | *SIR* |
| --- | --- | --- | --- |
| Con | 6.62±0.12 | 1190.5±211.7 | 0.62±0.077 |
| Ceph | 6.43±0.09 | 1161.9±174.1 | 0.57±0.023 |
| Pir | 6.56±0.11 | 1222.1±147.0 | 0.70±0.060 |

Table S2: Pre-incubation soil characteristics following 32-months of manure additions from either control cows (*Con*) or cows administered either the antibiotic cephapirin benzathine (*Ceph*) or pirlimycin hydrochloride (*Pir*). No significant differences between soil pH, 60-d carbon mineralization (*Cmin*) or active microbial biomass (*SIR*). Data are reported as means ± standard error.

Supplementary Table 3:

| Incubation  Temperature | Treatment | Post-Incubation  *SIR* | *∆SIR* | *Cmin* | *MSR* |
| --- | --- | --- | --- | --- | --- |
| 15 | *Con* | 0.51 ± 0.03 | -0.11 ± 0.06 | 626.4 ± 67.2 | 1112.2 ± 71.4 |
|  | *Ceph* | 0.44 ± 0.03 | -0.13 ± 0.04 | 560.0 ± 36.5 | 1103.4 ± 58.6 |
|  | *Pir* | 0.51 ± 0.03 | -0.20 ± 0.06 | 640.7 ± 31.8 | 1079.2 ± 50.4 |
| 20 | *Con* | 0.40 ± 0.03 | -0.22 ± 0.06 | 719.9 ± 90.4 | 1418.1 ± 108.4 |
|  | *Ceph* | 0.44 ± 0.02 | -0.13 ± 0.03 | 638.2 ± 39.0 | 1269.7 ± 79.4 |
|  | *Pir* | 0.46 ± 0.03 | -0.24 ± 0.05 | 688.1 ± 47.8 | 1190.5 ± 61.6 |
| 25 | *Con* | 0.43 ± 0.03 | -0.18 ± 0.09 | 1051.6 ± 49.5 | 2145.9 ± 198.7 |
|  | *Ceph* | 0.41 ± 0.01 | -0.16 ± 0.02 | 845.3 ± 55.9 | 1753.5 ± 156.0 |
|  | *Pir* | 0.4 ± 0.02 | -0.30 ± 0.07 | 826.7 ± 32.4 | 1517.4 ± 69.7 |
| 30 | *Con* | 1.00 ± 0.09 | 0.38 ± 0.11 | 1041.6 ± 52.8 | 1380.3 ± 118.9 |
|  | *Ceph* | 0.62 ± 0.05 | 0.05 ± 0.05 | 945.2 ± 33.7 | 1611.6 ± 60.1 |
|  | *Pir* | 0.57 ± 0.03 | -0.13 ± 0.07 | 973.0 ± 20.3 | 1558.9 ± 75.9 |

Table S3: Active microbial biomass as measured by substrate induced respiration (*SIR*; µg CO_2_-C g dry wt soil ^-1^ h^-1^) following the conclusion of the 60-d incubation (Post-incubation *SIR*), as well as the change in active microbial biomass from the beginning to the end of the incubation (*∆SIR*). Total carbon mineralized (*Cmin*) over the course of the 60-d incubation experiment. Mass-specific respiration (*MSR*; mg C-CO^2^ mg microbial biomass^-1^) measured during the 60-d incubation experiment. *MSR* is determined by the total respired carbon as a proportion of the active microbial biomass (*SIR*). *SIR* was estimated as an average of the starting and ending *SIR* measurements. Data are reported as mean ± standard error

Supplementary Table 4:

| Temperature | Treatment | Rhizobiales | Pedosphaerales | Myxococcales | Micropepsales | Gemmatales | Chthoniobacterales | Chitinophagales | Betaproteobacteriales | Acidobacteriales |
| --- | --- | --- | --- | --- | --- | --- | --- | --- | --- | --- |
| Temperature X Treatment Interaction | | *P* = 0.64 | *P* = 0.09 | *P* = 0.21 | *P* = 0.008 | *P* = 0.25 | *P* = 0.62 | *P* = 0.23 | *P* = 0.59 | *P* < 0.001 |
| 15 |  | *P* = 0.3 | *P* = 0.19 | *P* = 0.40 | *P* = 0.02 | *P* = 0.26 | *P* = 0.10 | *P* = 0.4 | *P* = 0.74 | *P* = 0.42 |
|  | Control |  |  |  | a |  |  |  |  |  |
|  | Cephapirin |  |  |  | b |  |  |  |  |  |
|  | Pirlimycin |  |  |  | ab |  |  |  |  |  |
| 20 |  | *P* = 0.61 | *P* = 0.55 | *P* = 0.1 | *P* = 0.06 | *P* = 0.97 | *P* = 0.79 | *P* = 0.63 | *P* = 0.04 | *P* = 0.31 |
|  | Control |  |  |  | a |  |  |  | ab |  |
|  | Cephapirin |  |  |  | b |  |  |  | a |  |
|  | Pirlimycin |  |  |  | ab |  |  |  | b |  |
| 25 |  | *P* = 0.64 | *P* = 0.008 | *P* = 0.02 | *P* < 0.001 | *P* = 0.22 | *P* = 0.03 | *P* = 0.26 | *P* = 0.08 | *P* < 0.001 |
|  | Control |  | a | ab | a |  | a |  |  | a |
|  | Cephapirin |  | b | a | b |  | ab |  |  | b |
|  | Pirlimycin |  | b | b | c |  | b |  |  | c |
| 30 |  | *P* = 0.14 | *P* = 0.03 | *P* = 0.24 | *P* < 0.001 | *P* = 0.16 | *P* < 0.001 | *P* = 0.46 | *P* = 0.05 | *P* < 0.001 |
|  | Control |  | a |  | a |  | a |  | a | a |
|  | Cephapirin |  | ab |  | b |  | b |  | b | b |
|  | Pirlimycin |  | b |  | b |  | b |  | ab | b |

Table S4: Results of the linear mixed effects models analyzing the difference in the relative abundance of the 10 most abundant bacterial orders. Initial full models were run to examine the influence of fixed effects and their interactions. Because of the significant interaction between treatment and temperature in our PERMANOVA analysis, we subsetted our data and examined the treatment effect at each incubation temperature. The significant values of these models are provided below the full interaction model. Letters denote significant differences among the treatments at each temperature.

Supplementary Table 5:

| Temperature | Treatment | Agaricales | Archaeorhizomycetales | GS11 | Helotiales | Hypocreales | o__Microascales | o__Mortierellales | o__Olpidiales | o__Pleosporales | Sordariales |
| --- | --- | --- | --- | --- | --- | --- | --- | --- | --- | --- | --- |
| Temperature X Treatment Interaction | | *P* = 0.137 | *P* = 0.3 | *P* < 0.001 | *P* = 0.12 | *P* = .25 | *P* < 0.001 | *P* = 0.033 | *P* = 0.148 | *P* = 0.62 | *P* = 0.297 |
| 15 |  | *P* = 0.668 | *P* = 0.19 | *P* = 0.46 | *P* = 0.21 | *P* = 0.265 | *P* = 0.35 | *P* = 0.77 | *P* = 0.127 | *P* = 0.486 | *P* = 0.84 |
|  | Control |  |  |  |  |  |  |  |  |  |  |
|  | Cephapirin |  |  |  |  |  |  |  |  |  |  |
|  | Pirlimycin |  |  |  |  |  |  |  |  |  |  |
| 20 |  | *P* = 0.28 | *P* = 0.33 | *P* = 0.40 | *P* = 0.18 | *P* = 0.07 | *P* = 0.29 | *P* = 0.59 | *P* = 0.67 | *P* = 0.36 | *P* = 0.66 |
|  | Control |  |  |  |  |  |  |  |  |  |  |
|  | Cephapirin |  |  |  |  |  |  |  |  |  |  |
|  | Pirlimycin |  |  |  |  |  |  |  |  |  |  |
| 25 |  | *P* = 0.18 | *P* = 0.002 | *P* < 0.001 | *P* = 0.08 | *P* = 0.02 | *P* = 0.03 | *P* < 0.001 | *P* = 0.007 | *P* = 0.57 | *P =* 0.04 |
|  | Control |  | a | a |  |  | a | a | A |  | Ab |
|  | Cephapirin |  | a | a |  |  | b | B | A |  | A |
|  | Pirlimycin |  | b | b |  |  | ab | B | b |  | B |
| 30 |  | *P* = 0.17 | *P* = 0.03 | *P* < 0.001 | *P* = 0.29 | *P* < 0.001 | *P* < 0.001 | *P* = 0.01 | *P* = 0.17 | *P* = 0.79 | *P =* 0.02 |
|  | Control |  | a | A |  | A | a | A |  |  | A |
|  | Cephapirin |  | a | A |  | B | b | Ab |  |  | Ab |
|  | Pirlimycin |  | b | b |  | ab | b | B |  |  | B |

Table S5: Results of the linear mixed effects models analyzing the difference in the relative abundance of the 10 most abundant fungal orders. Initial full models were run to examine the influence of fixed effects and their interactions. Because of the significant interaction between treatment and temperature in our PERMANOVA analysis, we subsetted our data and examined the treatment effect at each incubation temperature. The significant values of these models are provided below the full interaction model. Letters denote significant differences among the treatments at each temperature.

Figure S1


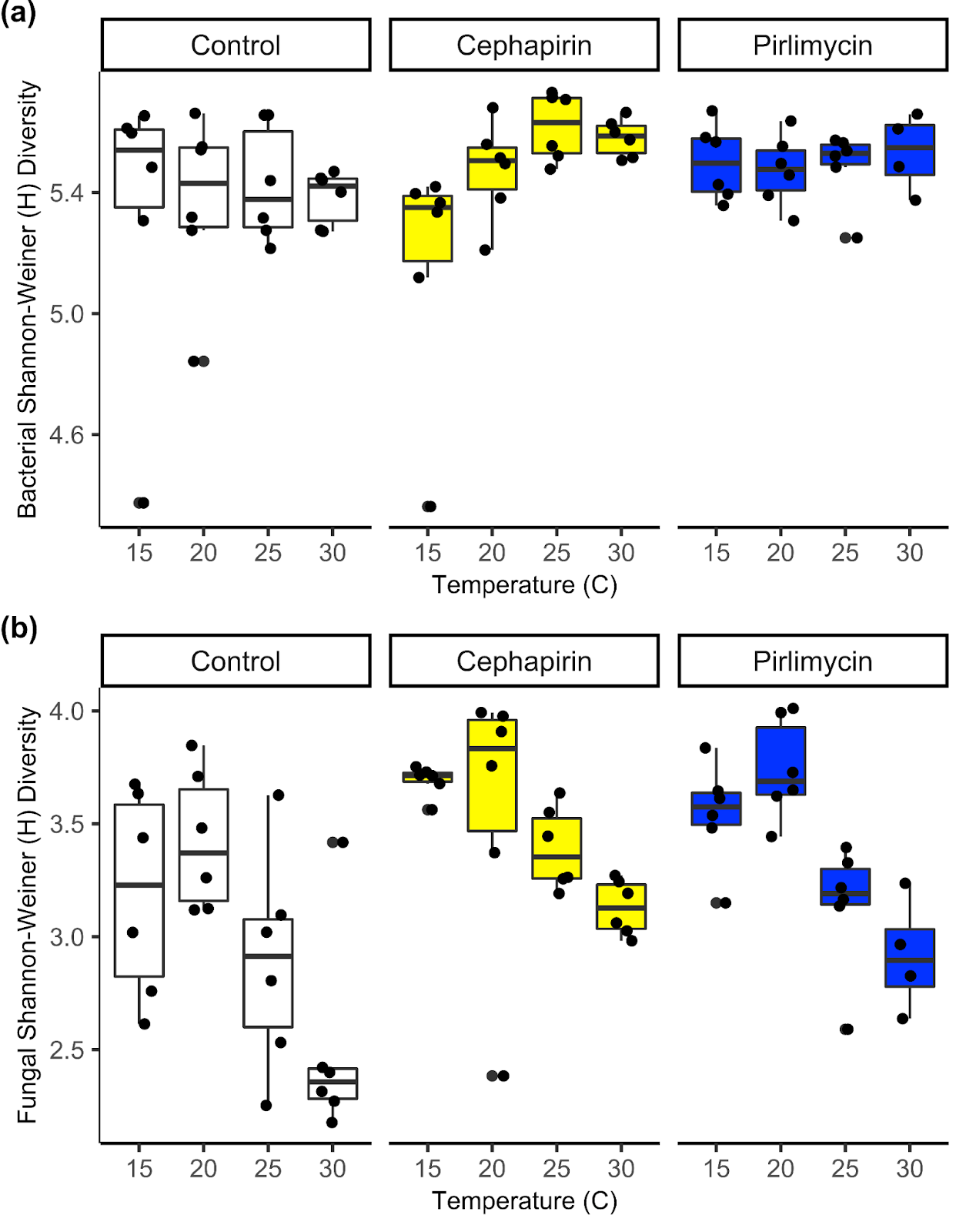


Figure S1:

Box plots of the average Shannon-Weiner (H) diversity response for bacterial (a) and fungal (b) and communities. Boxes are colored according to the antibiotic treatment, and temperatures are represented across the x-axis. The boxes represent the interquartile range (IQR) between the first and third quartiles (25 th and 75 th percentiles, respectively) and the vertical line inside the box defines the median. Whiskers represent the lowest and highest values within 1.5 times the IQR from the first and third quartiles, respectively, and outliers are represented as points outside the whiskers.

Figure S2:


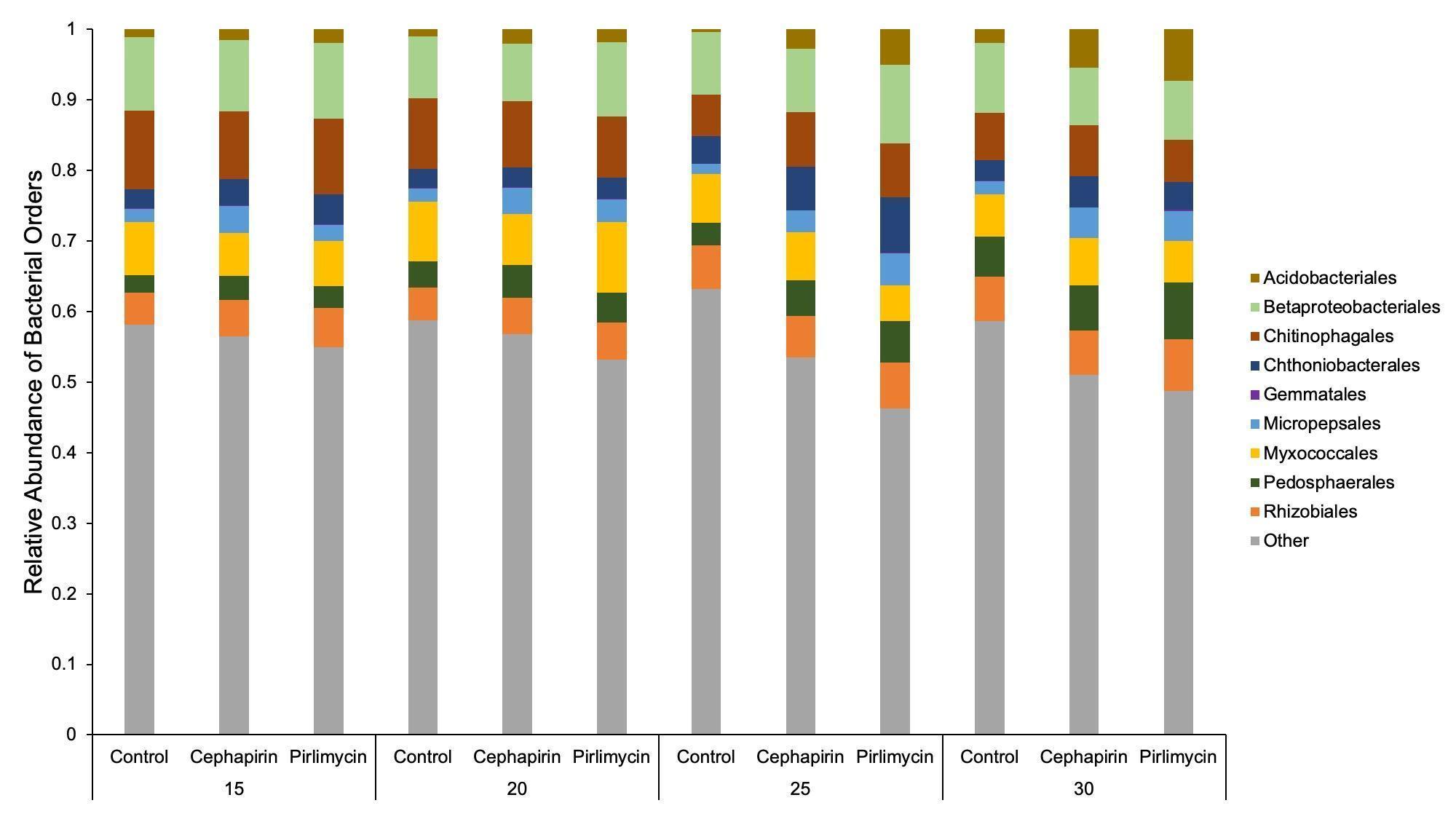


Figure S2: Stacked bar charts representing the relative abundance of the 10 most abundant orders of bacteria across treatments and temperatures.

Figure S3:


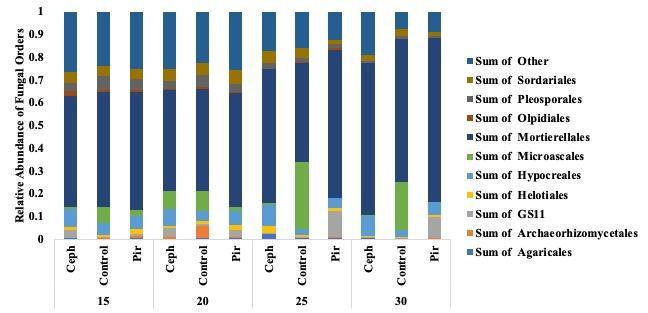

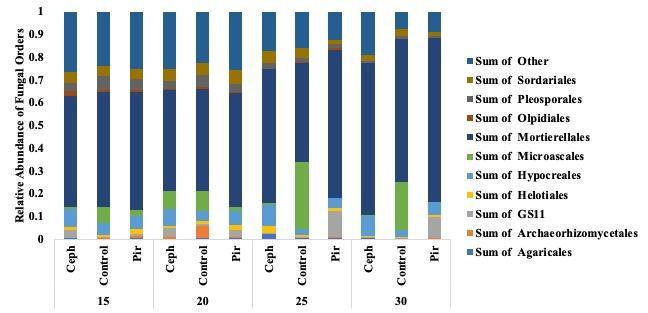

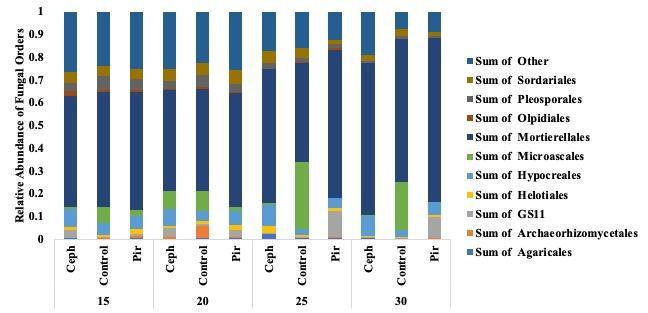


Figure S3: Stacked bar charts representing the relative abundance of the 10 most abundant orders of fungi across treatments and temperatures.
